# Methodological Considerations in Assessing Interlimb Coordination on Poststroke Gait: A Scoping Review of Biomechanical Approaches and Outcomes

**DOI:** 10.3390/s22052010

**Published:** 2022-03-04

**Authors:** Ana G. B. Couto, Mário A. P. Vaz, Liliana Pinho, José Félix, Sandra Silva, Augusta Silva, Andreia S. P. Sousa

**Affiliations:** 1 Escola Superior de Saúde de Santa Maria, Travessa Antero de Quental, 173, 4049-024 Porto, Portugal; ana.couto@santamariasaude.pt; 2 Centro de Investigação em Reabilitação, Centro de Estudos de Movimento e Atividade Humana, Rua Dr. António Bernardino de Almeida, 400, 4200-072 Porto, Portugal; liliana.pinho@ipsn.cespu.pt; 3 Faculdade de Engenharia, Universidade do Porto, Rua Dr. Roberto Frias, s/n, 4200-465 Porto, Portugal; 4 Departamento de Engenharia de Mecânica, Faculdade de Engenharia, Universidade do Porto (INEGI/Labiomep), Rua Dr. Roberto Frias, s/n, 4200-465 Porto, Portugal; gmavaz@fe.up.pt; 5 Escola Superior de Saúde do Vale do Ave, Cooperativa de Ensino Superior Politécnico e Universitário, Rua José António Vidal, 81, 4760-409 Vila Nova de Famalicão, Portugal; sandra.silva@ipsn.cespu.pt; 6 Faculdade de Desporto, Universidade do Porto, Rua Dr. Plácido da Costa, 91, 4200-450 Porto, Portugal; 7 Departamento de Física, Centro de Investigação em Reabilitação, Centro de Estudos de Movimento e Atividade Humana, Escola Superior de Saúde do Porto, Instituto Politécnico do Porto, Rua Dr. António Bernardino de Almeida, 400, 4200-072 Porto, Portugal; josefelixfelix15@gmail.com; 8 Área Científica de Fisioterapia, Centro de Investigação em Reabilitação, Centro de Estudos de Movimento e Atividade Humana, Escola Superior de Saúde do Porto, Instituto Politécnico do Porto, Rua Dr. António Bernardino de Almeida, 400, 4200-072 Porto, Portugal; smaugusta@gmail.com

**Keywords:** double support, interlimb, lower limbs, poststroke adults, walking

## Abstract

Objective: To identify and summarize biomechanical assessment approaches in interlimb coordination on poststroke gait. Introduction: Interlimb coordination involves complex neurophysiological mechanisms that can be expressed through the biomechanical output. The deepening of this concept would have a significant contribution in gait rehabilitation in patients with an asymmetric neurological impairment as poststroke adults. Inclusion criteria: Poststroke adults (>19 years old), with assessment of interlimb coordination during gait, in an open context, according to the Population, Concept, Context framework. Methods: A literature search was performed in PubMed, Web of Science™, Scopus, and gray literature in Google Scholar™, according to the PRISMA-ScR recommendations. Studies written in Portuguese or English language and published between database inception and 14 November 2021 were included. Qualitative studies, conference proceedings, letters, and editorials were excluded. The main conceptual categories were “author/year”, “study design”, “participant’s characteristics”, “walking conditions”, “instruments” and “outcomes”. Results: The search identified 827 potentially relevant studies, with a remaining seven fulfilling the established criteria. Interlimb coordination was assessed during walking in treadmill (*n* = 3), overground (*n* = 3) and both (*n* = 1). The instruments used monitored electromyography (*n* = 2), kinetics (*n* = 2), and kinematics (*n* = 4) to assess spatiotemporal parameters (*n* = 4), joint kinematics (*n* = 2), anteroposterior ground reaction forces (*n* = 2), and electromyography root mean square (*n* = 2) outcomes. These outcomes were mostly used to analyze symmetry indices or ratios, to calculate propulsive impulse and external mechanical power produced on the CoM, as well as antagonist coactivation. Conclusions: Assessment of interlimb coordination during gait is important for consideration of natural auto-selected overground walking, using kinematic, kinetic, and EMG instruments. These allow for the collection of the main biomechanical outcomes that could contribute to improve better knowledge of interlimb coordination assessment in poststroke patients.

## 1. Introduction

Interaction between posture and movement during walking [1,2] requires a close coordination of muscle activity between the two lower limbs [3] in a complex control of a moving center of mass (CoM), which is not within the base of foot support [1,2]. Defined as the timing of lower limbs motor cycles in one relative to the other [4], interlimb coordination reflects the principles of central networks organization to generate muscle activity patterns, that determine body kinematics, kinetics, and efficiency [5]. In fact, biomechanical factors [6,7,8,9], which seem to reflect both spinal [10,11,12,13,14] and supraspinal mechanisms [13,14,15,16,17,18,19] involved in interlimb coordination, demonstrate its strong association with gait economy [6,7,8,20].

From the first step, the central nervous system (CNS) seeks to achieve the perfect coordination of body segments to maintain postural stability during forward progression [9]. The relation between movement and postural control, depending, respectively, on the activation of the dorsolateral and ventromedial systems, is expressed through the functional unit composed by both lower limbs during walking to keep the body’s CoM over the feet [3]. This is particularly critical for stable human bipedal walking during step-to-step transition, considering its impact on the mechanical work on gait [7,8].

During the double support of walking, lower limb forces are needed to redirect the CoM velocity from a downward and forward direction to an upward and forward direction [6,8]. Particularly, the interruption of the energy-conserving motion during single support by an inelastic collision of the swing leg with the ground is related to negative work on the CoM that leads to changes in velocities of the lower limbs and the CoM [8,21]. Consequently, a substantial amount of positive mechanical work, predominantly related to the propulsion impulse in the trailing limb immediately before collision of the leading limb, is needed to redirect the CoM between steps [5,6,7,8,20,22]. Transition between steps reaches an optimum level when the terminal stance propulsion and the initial contact-loading response have the same magnitude and a short duration [8].

Gait disorders affects a large proportion of subjects after stroke and vary according to stroke severity, location of infarct and time since stroke [23]. Epidemiological data show that stroke lesion occurs predominantly in the middle cerebral artery territory which, by supplying both cortical and subcortical structures [24,25], compromise the contralesional dorsolateral and ipsilesional ventromedial systems [5]. As a consequence, the motor impairment may also arise on the ipsilesional side of the body, associated with a dysfunctional bilateral postural control, but not as severe as on the contralesional side [26,27]. This bilateral involvement sustains the decreased interlimb coordination demonstrated in post-stroke subjects [23,28]. This has been expressed through an asymmetric lower limb behavior [23,28], impairments in the step transition from one limb to the other [7,29], and leads to an increase of gait energy expenditure [30].

Considering that human walking is controlled by neural motor commands, through a complex orchestration of muscle forces and joint motions, several biomechanical variables, including electromyographic (EMG) activity, ground reaction forces (GRF), and resulting limb motions have been used to assess interlimb coordination during walking.

However, to the best of our knowledge, there is no consensus regarding the method and its suitability for assessing gait interlimb coordination in poststroke patients [31].

Thus, the purpose of this study was to review and summarize the methods/strategies used to assess the interlimb coordination, for a better understanding of its underpinning mechanisms, and its role in gait performance and rehabilitation in poststroke patients.

Review Questions

The two main questions which have been addressed in this review are:

i. Which walking conditions have been considered to assess the interlimb coordination in poststroke adults?
ii. What are the main instruments and outcomes that have been used to assess the interlimb coordination in poststroke adults?

## 2. Inclusion Criteria

Eligibility criteria were established a priori using the acronym PCC (Population, Concept, Context), defining the search strategy [32] as:

Population

Poststroke adults (>19 years old) [33].

Concept

Interlimb coordination assessment during gait.

Context

Open.

Types of source evidence

Studies written in Portuguese or English on poststroke adult population assessed in terms of interlimb coordination during gait were included. Qualitative studies, conference proceedings, letters, and editorials were excluded.

## 3. Methods

The scoping review was conducted in accordance with the Preferred Reporting Items for Systematic Reviewers and Meta-Analysis extension for Scoping Reviews (PRISMA-ScR) [32].

### 3.1. Search Strategy

The review was performed, aiming to identify relevant evidence on the theme, by combining specific terms with the Boolean logic strategies in the following expression: (interlimb AND (walk* OR gait OR locomotion) AND (“stance phase” OR “double support” OR “support phase” OR “step-to-step”) AND (stroke OR “Cerebral Vascular Accident” OR CVA), in the electronic databases PubMed, Web of Science™, Scopus®, and gray literature in Google Scholar™, between the database inception and 14 November 2021.

### 3.2. Source of Evidence Screening and Selection

**Figure 1:**
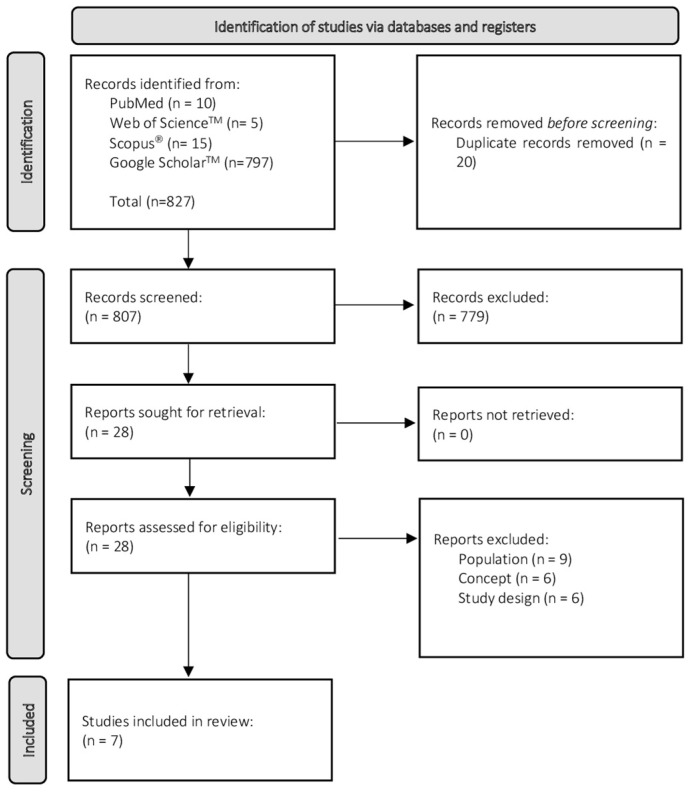
Flow diagram for the scoping review process adapted from the PRISMA statement [34].

The process of source of evidence screening and selection involved several stages. Elimination of duplicated records was automatically performed by the EndNote software. For the eligibility process, the search strategy was limited to titles and abstracts, and was performed by two independent reviewers (AGBC & LOP). This process was synthetized using worksheets of Excel 2021 (Microsoft^®^). The reference lists of all studies were also scanned to identify other potential eligible articles. When discrepancies occurred between reviewers on whether the study should be included in the review, the reasons for disagreement were analyzed, the trial report was consulted, and a consensus was achieved. This process consisted of a selection of 25 randomized titles/abstracts to consider; the independent reviewers screened the selected sample using the eligibility criteria and definitions/elaboration for this scope; they then met to discuss discrepancies, and made modifications to the eligibility criteria and definitions/elaboration to be analyzed. The reviewers only started screening when an agreement of 75% (or greater) was achieved. The results of the search were presented in a PRISMA-ScR flow diagram as shown is Figure 1.

### 3.3. Data Extraction

Data from publications were extracted based on the following main conceptual categories were “author/year”, “study design”, “participant’s characteristics”, “walking conditions”, “instruments”, and “outcomes”.

### 3.4. Analysis and Presentation of Results

The screening and selection process were presented in a diagrammatic form. A tabular form and a descriptive summary were used to describe the main conceptual categories, answering the review objectives and questions.

## 4. Results

The search identified 827 potentially relevant studies, 10 from PubMed, 5 from Web of Science™, 15 from Scopus^®^, and 797 from Google Scholar™. After removing 20 duplicated records, 807 remained, of which 779 were excluded after screening the title and abstract. Over the 28 lasting, 21 studies were excluded after full-text reading, as they did not address the proposed questions for this review. The remaining seven studies fulfilled the established criteria.

### 4.1. Study Design

Of the seven eligible studies, four studies included in this review were observational, transversal, analytical [35,36,37,38], two were quasi-experimental [39,40], and one was randomized controlled trial [41]. The publication year ranged from 2009 to 2016. It should be noted that older studies were published 12 years ago [36,40] (see Table 1).

### 4.2. Poststroke Participant’s Characteristics

In the seven studies included, three were performed exclusively with poststroke participants [36,37,41], and f included also healthy participants [35,38,39,40]. The sample sizes of poststroke participants varied from 10 [36] to 26 participants [37].

A total of 114 poststroke participants were included in the considered studies, totalizing 68 males and 46 females, with a mean age of 54.67 years old. Only two studies did not characterize anthropometric data of the sample, namely weight and height [40,41].

All included participants were in a chronic stage, ranging from 6 months poststroke [39] to 16.25 years [37], presenting a single and unilateral episode of stroke.

A total of four studies presented information about stroke type [35,38,39,40]: two studied ischemic and hemorrhagic stroke [39,40], and two analyzed only ischemic stroke [35,38]. According to lesion location, two studies defined the middle cerebral artery as the lesion area [35,38], and two described several specific stroke location [39,40]. Overall, three studies did not present information about stroke type or lesion location [36,37,41].

All studies were performed on left and right stroke participants, totalizing 65 left side strokes and 49 right side strokes.

To accomplish the understanding of following instructions in the requested tasks, three studies used mini-mental state examination to ensure it [35,38,41]. To assess sensorio-motor lower limb impairment, the Fugl–Meyer assessment was applied in five studies [35,37,38,39,40]. Other clinical measures were included in the two of the studies, namely the functional gait assessment, the activities-specific balance confidence questionnaire [39], and the Brunnström and Ashworth modified scale [41]. Only one study did not identify any clinical measure to accomplish eligibility criteria or sample characterization [36] (see Table 1).

### 4.3. Walking Conditions

A total of three studies assessed interlimb coordination during walking on the treadmill [37,39,41], using different speeds [37,39], or self-selected gait speed [41]. All three studies that assessed interlimb coordination during overground walking, used a self-selected gait speed [35,38,40]. Only one study included both treadmill and overground walking at a self-selected gait speed [36] (see Table 1).

### 4.4. Instruments

To explore interlimb coordination, several instruments were used to assess EMG, kinetics and kinematics data. In total, four studies included kinematic analysis [36,39,40,41], one only kinetic analysis [37], one kinetic and EMG analysis [38], and one only EMG analysis [35] (see Table 2).

To assess kinematic data, three different motion capture systems were used: OPTOTRACK [40], Vicon™ [36,39], and Bioengineering Tracker Analyser Soft (BTS) [41], with a number of cameras ranging from 5 [36] to 12 [39]. One study did not described the number of the cameras [40]. Reflective markers were located on lower limbs, pelvis, and sacrum in two studies [36,41], 1 study considered lower limbs, pelvis and shoulder [40] and one study only on feet and hands [39]. Within each identified segment, different anatomical references were considered, according to each author (see Table 2).

Kinetic data was evaluated through two Bertec force plates [37,38].

To assess EMG activity, a bioPLUX system was applied bilaterally to record data from several lower limb muscles: soleus, gastrocnemius medialis, and tibialis anterior in two studies [35,38], and rectus femoris, biceps femoris, and vastus medialis in one study [38] (see Table 2).

### 4.5. Main Outcomes and Significant Findings to Assess Interlimb Coordination, during Gait

Regarding kinematics, four studies explored symmetry of spatiotemporal parameters namely step length [36,40,41], double support time [36,39,40], stance time [36,41], step time and single support time [36], and swing time [41]. Only one of these considered interlimb symmetry ratio through the above spatiotemporal parameters [36]. Joint kinematics were studied by two authors considering the range of motion of hip and knee joints [36,41] and ankle joint [36] (see Table 2).

A total of two studies defined as kinetic outcome the integral of the anteroposterior component GRF, using different strategies to calculate propulsive impulse [37,38]. This impulse allowed to calculate the paretic propulsion in 1 study [37]. Still in kinetic context, one study referred the external mechanical power and step length asymmetry [37] (see Table 2).

Concerning EMG data, two studies contemplated the magnitude of the muscle activity through root mean square analysis [35,38]. This measure was used to be correlated with the propulsive impulse kinetic variable in one study [38], and to calculate antagonist coactivation ratio in another [35] (see Table 2).

## 5. Discussion

This scoping review gathered the literature that analyzed methods/strategies to assess interlimb coordination during gait on poststroke adults. Specifically, walking conditions, the main instruments used, and the related outcomes have been analyzed.

This information will allow a better understanding of the mechanisms underpinning interlimb coordination and its role in gait performance and rehabilitation, while its assessment promotes a reflection on key information to consider in future research.

### 5.1. Walking Conditions

According to the present review, interlimb coordination during gait was assessed in both treadmill [36,37,39,41] and overground walking [35,36,38,40]. Since there are different task demands and characteristics between treadmill and overground walking, these should be considered in the analysis of interlimb coordination.

Despite providing valuable information about interlimb coordination as a result of better monitoring conditions, walking on the treadmill may have some differences when comparing to individuals self-selected speed, in their natural walking [42]. While on a treadmill the surface is moving without changes in environment, static surface overground requires constant adaptation to it [40,43].

Kinematic and kinetic parameters [43,44] and muscle activity [44] were described with small differences when analyzed between treadmill and overground gait on healthy participants, suggesting qualitative and quantitative similarity. These small differences are generally within the normal variability of gait parameters [43], showing quite a similar behavior [44].

Puh and Baer [36] suggested with their study, on poststroke participants, that the treadmill training may be a useful tool to some areas of rehabilitation, but it cannot replace gait training overground. It may, however, be useful to use treadmill to focus on improving specific deficits, if combined with overground walking to ensure transferability of an improved gait pattern [36], such as an improvement on step length asymmetry, being more energetically advantageous [45]. From a therapeutic perspective, this seems to suggest that training individuals with neurological injuries on a treadmill appears to be justified [44].

Despite similarities overlap differences between treadmill and overground walking, interlimb coordination assessment, regarding poststroke participants, seems to be more suitable on overground context, considering individual previous accommodation and possible behavior changes on gait parameters when using treadmill. Overground walking may interfere less with natural characteristics of disabled participants [40].

### 5.2. Instruments

To evaluate kinematic data, three optoelectronic systems were used: OPTOTRACK [40], Vicon™ [36,39], and BTS [41]. These measurement systems appear to be the most accurate, and are described in literature as the gold standard in motion capture [46].

The accuracy of the systems depends on the cameras locations relative to each other, the distance between the cameras and the markers, the position, number, and type of the markers, and the motion of the markers within the capture volume [47], being that this one is dependent on the maximum number of cameras and the field of view of each camera [48].

Concerning the number of cameras, 5 [36] to 12 [39] were identified. Knowing that less cameras can interfere with marker gaps, using a smaller number might be seen as a disadvantage [49].

The optoelectronic systems found included active and passive marker systems. Vicon™ [36,39] and BTS [41] passive motion capture systems use markers that reflect light back to the sensor. Active marker optical systems, such as OPTOTRACK [40], utilize markers that contain the source of light for the sensors (often infrared) [50]. The benefit of active markers over passive ones could be the robustness of the measurements. However, active markers require additional cables and batteries, thus the freedom of movement is more limited [51].

Different models for motion capture were found, with differences in marker sets and protocols that details different number and position of markers. This fact can interfere in a large effect on the kinematic angles generated [52]. One of the biggest challenges in marker-based motion capture is maintaining accuracy and precision. Appropriate models for one’s specific research purpose and a single examiner precisely placing the markers may be important factors to maintain these assumptions [53].

Overall, to ensure the accuracy of the instruments, it seems important a description of all parameters worrying their well use. Moreover, to increase the robustness of the data and to keep up with technological developments, researchers could consider a prior test to experiment protocol and improve the most precise setups.

To evaluate kinetic data, two Bertec force plates were identified [37,38]. A force plate measures the GRF over time during static or dynamic motions such as gait. They provide information on the three-dimensional components of force (X, Y and Z), which can then be used to look at more in-depth joint loadings [54]. They are considered the gold standard model, with high accuracy, to determine spatiotemporal gait parameters related to GRF characteristics [54].

In this review, two authors resorted surface EMG to assess muscle activity during a dynamic task [35,38]. Surface EMG provides a non-invasive, global measurement of muscle activity, which may be more suitable for applications in movement analysis that require frequent assessments or information on the patterns of activation of multiple muscles [55,56,57]. Careful skin preparation and choice of electrode type, electrode placement, and recording configuration, including filter settings and amplifier gain, are essential to record high quality EMG signals with low noise [55,56,57].

To record this muscle activity, a bioPlux device was used [35,38]. The bioPlux is a device that collects and digitizes the signals from the electromyographic sensors transmitting them to a computer, where they are viewed in real time [58]. Despite its usefulness, the connection between the sensors and the equipment is ensured by cables that might interfere in movement freedom during the dynamic assessment. Nowadays, different type of solutions could be considered, using wireless technology, providing better data quality.

Several muscles were identified to evaluate interlimb coordination. The lower limb distal muscles were the most assessed in the included studies [35,38]. The number and selection of the assessed muscles might be justified by the number of channels available on the equipment used, as well as the specific research purpose of each study.

It is suggested that the surface EMG combined with kinematic and kinetic data is a useful tool for decision making of the appropriate method needed.

### 5.3. Main Outcomes and Significant Findings to Assess Interlimb Coordination, during Gait

To assess the interlimb coordination, several outcomes have been considered, including kinematics, kinetics, and EMG data.

A higher variety of kinematic outcomes was found, and the most pointed were related to spatiotemporal parameters as step length [36,40,41] and double support time [36,39,40]. Literature reports that asymmetry of step length following stroke is related to decreased propulsive force of the contralesional limb, decreased work and power of the contralesional plantar flexors, and decreased walking speed [59]. Moreover, asymmetric double support time is related to decreased speed [60]. In fact, according to the theoretical model in biomechanical study proposed by Kuo [8], the double support corresponds to step-to-step transition, and this transition from one stance limb inverted pendulum to the next appears to be the major determinant of the mechanical work of walking. Considering the aforementioned, the transition between steps reaches an optimum level when the propulsion of the trailing and the initial contact loading response of the leading have the same magnitude and short duration [8]. Thus, step length and double support time symmetry between both lower limbs appear to allow for a better understanding on interlimb coordination behavior.

Considering these assumptions, some authors also propose to combine the study of joint kinematics symmetry with spatiotemporal parameters, in order to improve this knowledge [36,41].

Regarding kinetic analysis, different outcomes were identified in this review: anteroposterior component GRF, to study the propulsive impulse [37,38], and the external mechanical power [37]. Despite some symmetry being required between lower limbs, the studies that evaluated the CoM mechanical work of leading and trailing showed that the trailing needs to generate more work than the leading one [38], probably through feedforward mechanism expressed through a lag between the peak of anteroposterior GRF trailing and leading limbs [61]. According to the theoretical model, approximately 75% of the energy loss during leading limb heel strike is compensated by the trailing limb during the propulsion in the double support phase [8,22].

Concerning EMG outcomes, muscle activity magnitude was expressed by RMS [35,38], allowing for the study of the correlation between lower limbs muscle activity and propulsive impulse [38]. This is supported by a previous study, on healthy subjects, showing a correlation between the EMG activity of the trailing limb and the magnitude of the leading anteroposterior GRF, during step-to-step transition, finding a higher correlation between them, which corroborates its major importance in forward propulsion [62].

Another outcome identified in this study results was the antagonist coactivation ratio [35], showing that the dynamic relationship between limbs during walking may also be analyzed through the levels of the antagonist coactivation ratio considering the functional position of each limb (trailing vs. leading) and the subsequent role of each.

Considering the exposed, it is suggested to assess interlimb coordination resorting to more than one outcome, through the combination of the different EMG, kinetic, and kinematic data.

Despite the importance of summarizing the main parameters to be considered in the interlimb coordination, it is not only enough the identification of the better outcomes to consider. It is also important to understand their behavior and to know their psychometric characteristics to find the better ones to study interlimb coordination, and allowing for monitoring of clinical improvements.

## 6. Conclusions

Since gait is a functional task from the perspective of rehabilitation, natural auto-selected walking should be pondered.

Kinematic, kinetic and EMG were assessment approaches found, allowing for the study of spatiotemporal parameters and joint kinematics, anteroposterior GRF, and magnitude of EMG activity. Through these biomechanical outcomes, it would be possible to highlight, as significant findings to assess interlimb coordination, the symmetry indices between lower limbs, propulsive impulse, and the external mechanical power produced on the CoM of each lower limb, and the antagonist coactivation ratio during gait in poststroke patients.

This review could improve a better understanding of the mechanisms supporting interlimb coordination assessment, as well as its role in poststroke gait performance and rehabilitation.

## Figures and Tables

**Table 1:** Descriptive poststroke participant´s characteristics and walking conditions descriptions.

Author, Year	Study Design	Poststroke Participant’s Characteristics		Walking Conditions
Reisman et al., 2009	Quasi-experimental	*n* = 11 (2 F, 9 M) Age: 55 (35–70) years old Stroke characteristics Time poststroke onset: (8–128) months Stroke side: left (6), right (5) Stroke type: ischemic (7), hemorrhagic (4) Stroke localization: parietal (2), hemisphere (6), basal ganglia (1), caudate head, anterior limb internal capsule (1), posterior temporoparietal (1) Clinical measures Lower Extremity Fugl–Meyer Assessment		Overground walking Self-selected speed
Puh & Bear, 2009	Observational, transversal, analytical	*n* = 10 (5 F, 5 M) Age: 37.3+/−10.6 (19–54) years old Height: 171.5+/−7.2 (163–185) cm Body weight: 71.5+/−14.2 (53.5–100) kg Stroke characteristics Time poststroke onset: 26+/−19.6 (9–95) months Hemiparetic side: left (2), right (8)		Overground walkingand treadmill walking Self-selected speed
Sousa et al., 2013	Observational, transversal, analytical	*n* = 16 (8 F, 8 M) Age: 53.87+/−7.17 years old Height: 1.65+/−0.10 m Body weight: 75.29+/−7.03 kg Stroke characteristics Time poststroke onset: 26+/−9 months Stroke type: ischemic (16) Stroke side: left (11), right (5) Stroke localization: middle cerebral artery Clinical measures Lower Extremity Fugl–Meyer Assessment Mini-Mental State Examination		Overground walking Self-selected speed
Krasovsky et al., 2013	Quasi-experimental	*n* = 10 (0 F, 10 M) Age: 60.3+/−11.3 years old Height: 176.3+/−8.4 cm Body weight: 87.1+/−13.5 kg Stroke characteristics Time poststroke onset: 28.9+/−23.4 months Stroke type: ischemic (8), hemorrhagic (2) Stroke side: left (4), right (6) Stroke localization: middle cerebral artery-internal capsule (1), middle cerebral artery (2), bulbar (1), caudate nucleus (1), thalamus-internal capsule (1), subcortical (2), anterior choroidal artery (1), putamen-internal capsule (1) Clinical measures Functional Gait Assessment Activities-Specific Balance Confidence Questionnaire Lower Extremity Fugl–Meyer Assessment		Treadmill walking Conditioned at different speeds
Mahon et al., 2015	Observational, transversal, analytical	High-speed group *n* = 13 (6 F, 7 M) Age: 56+/−8.4 years old Height: 175+/−8.4 cm Body weight: 91+/−18 kg Stroke characteristics Time poststroke onset: 103+/−92 months Hemiparetic side: left (7), right (6) Clinical measures Lower Extremity Fugl–Meyer Assessment	Low-speed group *n* = 13 (6 F, 7 M) Age: 54+/−12 years old Height: 173+/−9.3 cm Body weight: 93+/−13 kg Stroke characteristics Time poststroke onset: 30+/−17 months Hemiparetic side: left (7), right (6) Clinical measures Lower Extremity Fugl–Meyer Assessment	Treadmill walking Conditioned at different speeds
Silva et al., 2015	Observational, transversal, analytical	*n* = 16 (8 F, 8 M) Age: 53.87+/−7.17 years old Height: 1.65+/−0.10 m Body weight: 75.29+/−7.03 kg Stroke characteristics Time poststroke onset: 26+/−9 months Stroke type: ischemic (16) Stroke side: left (11), right (5) Stroke localization: middle cerebral artery Clinical measures Lower Extremity Fugl–Meyer Assessment Mini-Mental State Examination		Overground walking Self-selected speed
Drużbicki et al., 2016	Randomized Controlled Trial	Intervention group *n* = 15 (6 F, 9 M) Age: 61.9+/−11.4 years old Stroke characteristics Time poststroke onset: 36 (8–120) months Hemiparetic side: left (6), right (9) Clinical measures Mini-Mental State Examination Brunnström Ashworth Modified Scale	Control group *n* = 10 (5 F, 5 M) Age: 59.8+/−11.7 years old Stroke characteristics Time poststroke onset: 38.2 (8–110) months Hemiparetic side: left (6), right (4) Clinical measures Mini-Mental State Examination Brunnström Ashworth Modified Scale	Treadmill walking Self-selected speed

cm: centimeter; F: Female; kg: kilogram; M: Male; m: meter.

**Table 2:** Instruments, main outcomes, and significant findings to assess interlimb coordination, during gait.

Author, Year	Instruments	Main Outcomes	Significant Findings to Assess Interlimb Coordination
Reisman et al., 2009	Kinematic OPTOTRACK (Northern Digital, Waterloo, ON, Canada) Infrared emitting diodes: foot (fifth metatarsal head), ankle (lateral malleolus), knee (lateral joint space), hip (greater trochanter), pelvis (iliac crest), and shoulder (acromion process), bilaterally	Spatiotemporal parameters: Step length (m) Double support time (% gait cycle)	Interlimb symmetry of the spatiotemporal parameters
Puh & Bear, 2009	Kinematic ViconTM 370 5 cameras Reflective markers: second sacral vertebra, on the anterior superior iliac spine, the femoral epicondyle, lower lateral surface of the thigh and calf, lateral malleolus, calcaneus, and the second metatarsal head, bilaterally	Spatiotemporal parameters: Step length (m) Step time (s) Stance time (absolute (s) and relative (% gait cycle)) Single support time (absolute (s) and relative (% gait cycle)) Double support time (absolute (s) and relative (% gait cycle)) Joint kinematics: Ankle, knee and hip (^o^)	Interlimb symmetry of the spatiotemporal parameters using the formula: Interlimb symmetry ratio = 2NHP/(NHP + HP) Interlimb symmetry of the mean joint kinematics amplitude
Sousa et al., 2013	Kinetic 2 force plates (Bertec Corp, 6171 Huntley Rd, Ste J, Columbus, OH 43229; models FP4060−10 and FP4060−08) Electromyography bioPLUX (PLUX Wireless Biosignals, Arruda dos Vinhos, Portugal) Bilateral lower limbs EMG activity: SOL, GASm, TA, BF, RF and VM	Ground reaction force: Integral Fy Electromyograpic activity: Magnitude by root mean square (mV)	Percentage of the propulsive impulse = ∫Fy_trail_/(∫Fy_trail_ + ∫Fy_lead_) × 100 Statistic analisys through a correlation coefficient: Relation between contralesional limb electromyography and ipsilesional limb propulsive/braking impulse
Krasovsky et al., 2013	Kinematic ViconTM 512 12 cameras Reflective markers: back of the heel, on the forefoot (head of the 2nd metatarsal), and on the hand (head of 2nd metacarpal), bilaterally	Temporal parameters: Double support time (s)	Interlimb symmetry of the double support duration
Mahon et al., 2015	Kinetic 2 force plates (Bertec Corporation, Columbus, Ohio)	Ground reaction force: Integral Fy Spatial parameters: Step length	Individual limb mechanical power on the CoM Propulsive impulse = Integral of positive Fy over a complete stride (HP and NHP) Paretic propulsion = PI HP/(PI HP + PI NHP) Step length asymmetry = Max NHP + Max HP/(NHP + HP)
Silva et al., 2015	Electromyography bioPLUX (PLUX Wireless Biosignals, Arruda dos Vinhos, Portugal) Bilateral activity of the ankle agonist muscles: TA in the lead limb and GASm, SOL in the trail limb	Electromyograpic activity: Magnitude by root mean square (mV)	Ankle antagonist coactivation (%) = (antagonist activity/(agonist + antagonist activity)) × 100
Drużbicki et al., 2016	Kinematic Bioengineering Tracker Analyzer Software 6 infrared cameras Reflective markers (internal protocol of the system Helen Hayes (Davis) Marker Placement): sacrum, pelvis (anterior posterior iliac spine), femur (lateral epicondyle, greater trochanter and in lower 1/3 of the shank), fibula (lateral malleolus, lateral condyle and in lower 1/3 of the shank), and foot (metatarsal head and heel), bilaterally	Spatiotemporal parameters: Step length (m) Stance time (s) Swing time (s) Joint kinematics: Knee and hip (^o^)	Interlimb symmetry index for spatiotemporal parameters and mean joint kinematics amplitude: = [(paretic − non-paretic)/0.5(paretic + non-paretic)] × 100% Interlimb symmetry ratio: = paretic/non-paretic of the spatiotemporal parameters and mean joint kinematics amplitude

BF: Biceps Femoris; CoM: center of mass; EMG: electromyographic; Fy: anteroposterior ground reaction force; GASm: Gastrocnemius medialis; HP: hemiplegic; m: meters; Max: maximum; mV: millivolts; NHP: non-hemiplegic; PI: propulsive impulse; RF: Rectus Femoris; s: seconds; SOL: Soleus; TA: Tibialis Anterior; VM: Vastus Medialis.

## Data Availability

Not applicable.
